# Supplementary material for: Characterization of a Programmable Argonaute Nuclease from the Mesophilic Bacterium Rummeliibacillus suwonensis
Source: Biomolecules. 2022 Feb 23;12(3):355. doi: 10.3390/biom12030355 (PMC8945025; doi:10.3390/biom12030355)
Supplement: Supplementary file 1 [file biomolecules-12-00355-s001.zip › biomolecules-1533238-supplementary final .pdf]

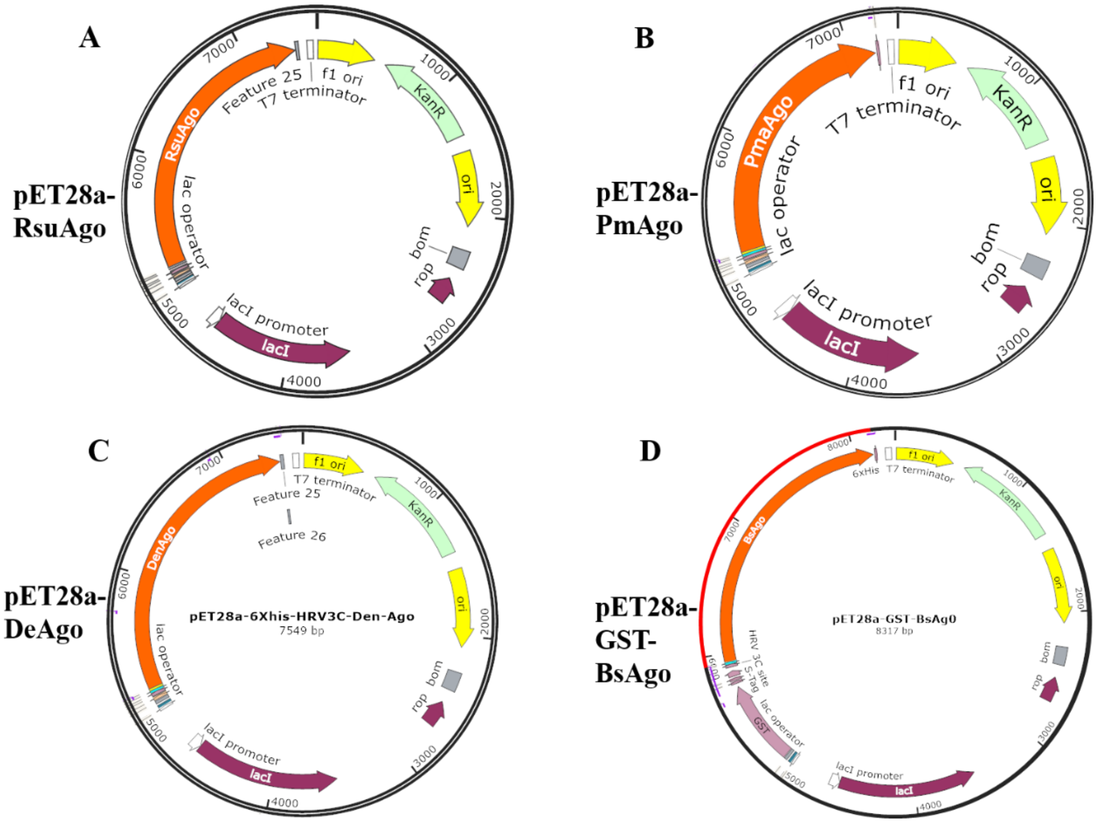

Figure S1. Schematic representation of the pET28a-based expression vector. (**A**) pET28a-RsuAgo (**B**) pET28a-PmAgo (**C**) pET28a-DeAgo (**D**) pET28a-GST-BsAgo. A His(6x)-GST tag was N-terminally fused to BsAgo linked with a cleavable HRV 3C site. All pAgos were under the control of a T7 promoter.

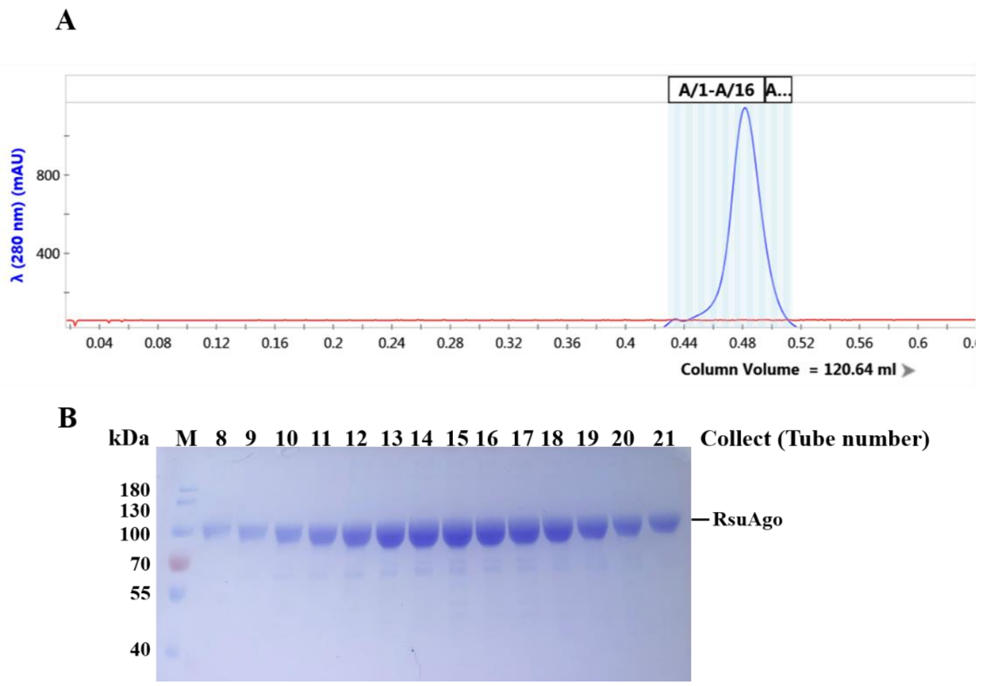

Figure S2. Purification of RsuAgo protein. (**A**) Size exclusion diagram showing the elution peak of

RsuAgo. (**B)** The purity of the purified RsuAgo was determined using SDS-PAGE. M, marker.

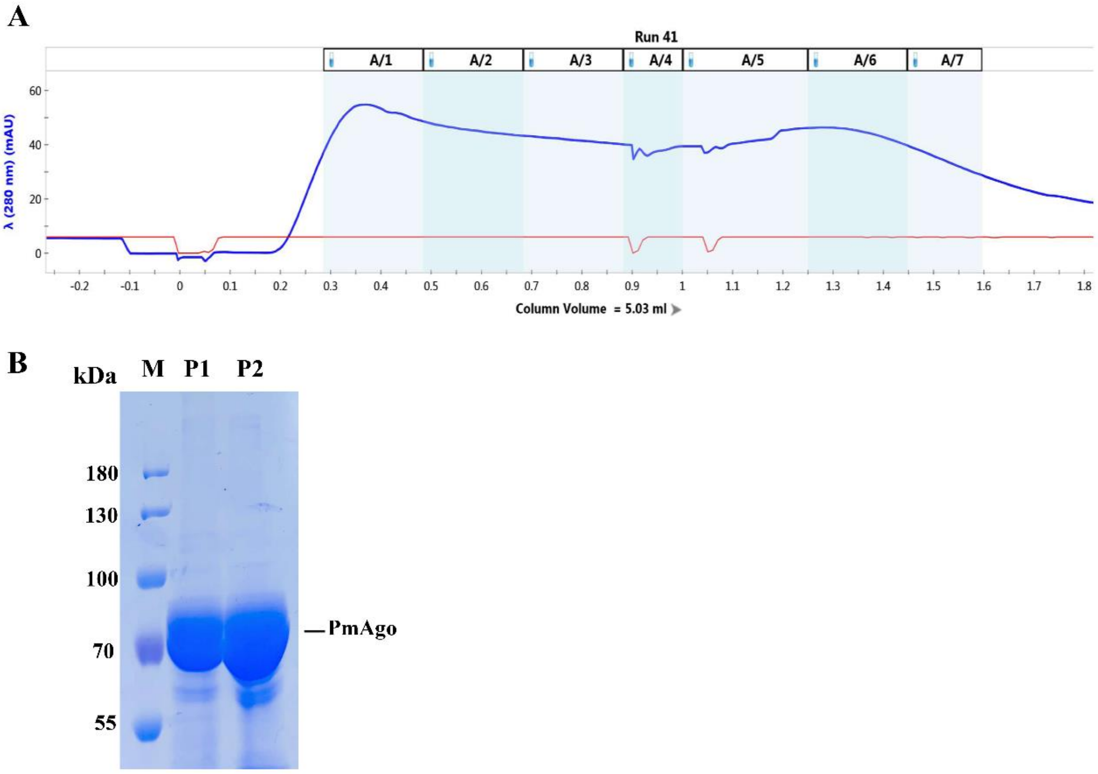

Figure S3. Purification of PmAgo protein. (**A)** Heparin column affinity chromatography diagram showing the elution peak of PmAgo. (**B)** The purity of the purified PmAgo was determined using SDS-PAGE. M, marker; P1, two-step heparin affinity-purified protein lane; P2, one-step Ni-NTA purified PmAgo protein lane.

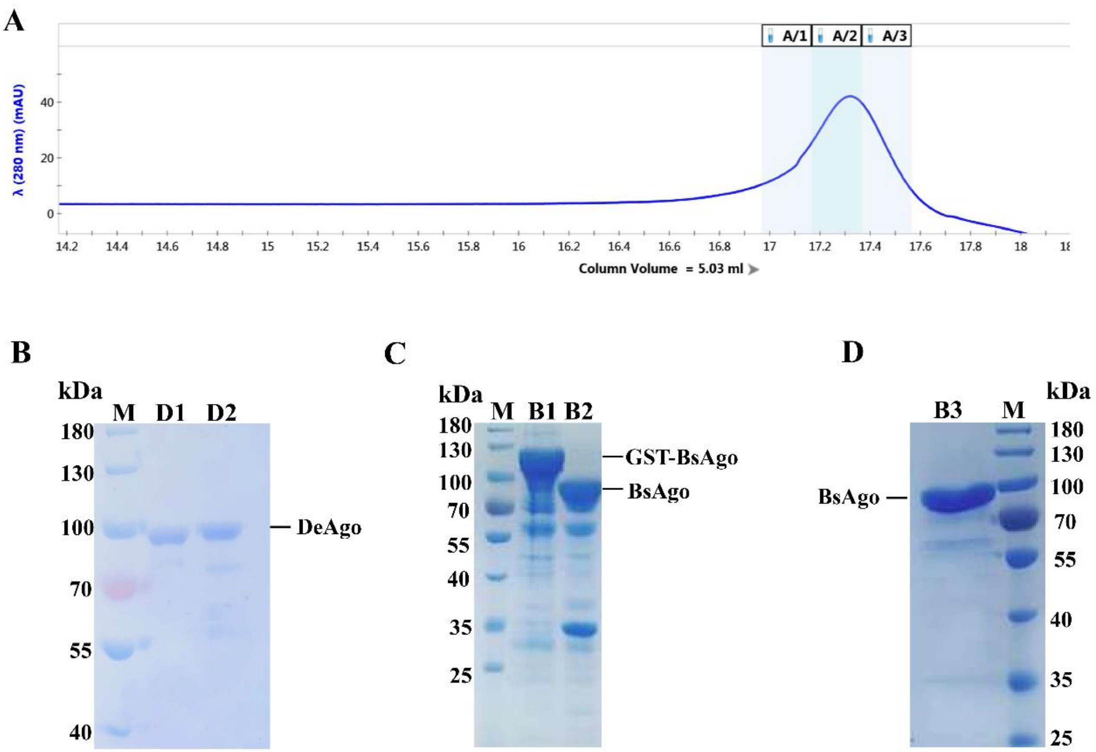

Figure S4. Purification of DeAgo and BsAgo protein. (**A**) Heparin column affinity chromatography diagram showing the elution peak of DeAgo. (**B**) The purity of the purified DeAgo was determined using SDS-PAGE. D1, one-step Ni -NTA purified pAgo protein lane; D2, two-step heparin affinity-purified protein lane. (**C**) 6 × His tag and GST tag were removed by cleavage with HRV 3C overnight at 4 °C and the cleaved protein was separated from the fusion tag using a second Ni-NTA affinity step. B1, uncleaved GST-BsAgo protein lane; B2, cleaved BsAgo protein lane. **(D)** Two Ni-NTA purified BsAgo was determined using SDS-PAGE. B3, BsAgo concentrated by Amicon 50K filter unit (Millipore) protein lane; M, marker.

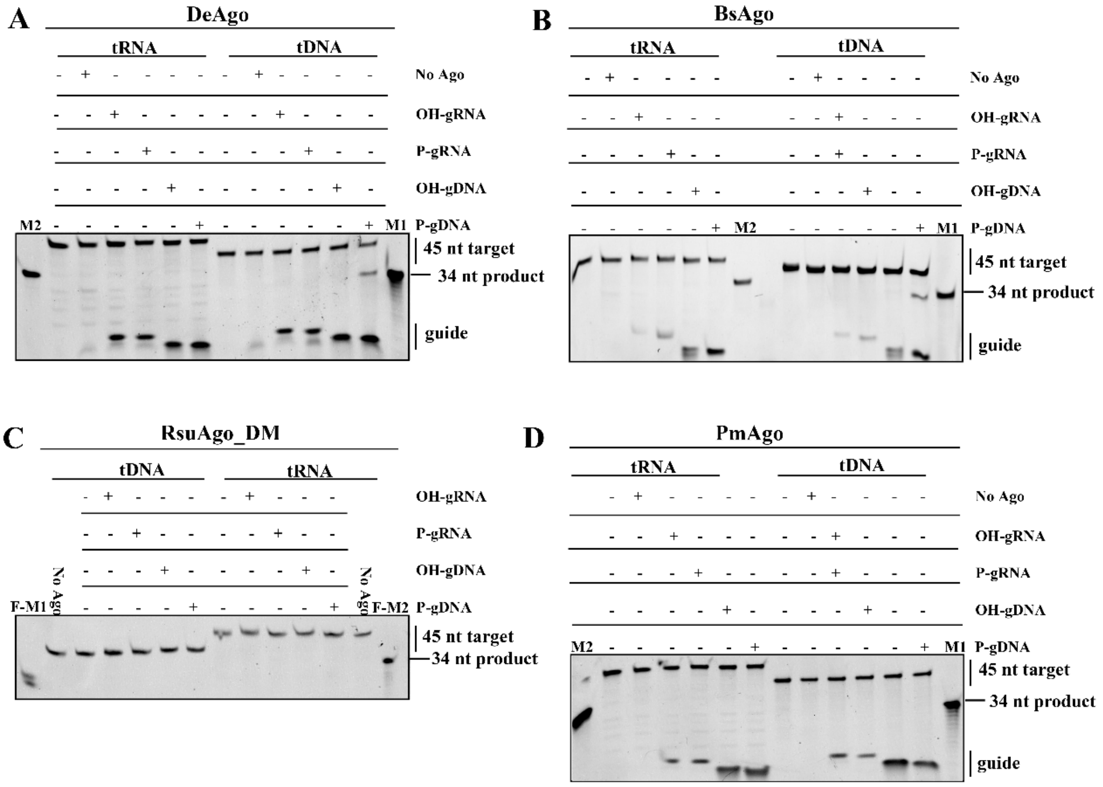

Figure S5. Analysis of single-stranded nucleic acid cleavage activity of several other pAgos . **(A)** Cleavage by DeAgo with no FAM-labeled DNA and RNA targets. (**B)** Cleavage by BsAgo with no FAM-labeled DNA and RNA targets. (**C)** Cleavage by RsuAgo_DM with FAM-labeled DNA and RNA targets. (**D)** Cleavage by PmAgo with no FAM-labeled DNA and RNA targets. F-M1 (or M1) and F-M2 (or M2) are chemically synthesized, 34 nt long ssDNA and ssRNA oligonucleotides with a FAM-label (or no FAM-label) at the 5'-end that were loaded on gels as size markers corresponding to the length of the DNA and RNA cleavage products, respectively. For single-stranded nucleic acid cleavage by RsuAgo_DM, FAM-labeled marker was used and the FAM-labeled target was added, then the denaturing PAGE gels were directly visualized with gel Doc^{TM} XR+ (Bio-Rad) without being stained. All experiments were performed at the 4: 2: 1 RsuAgo: guide: target molar ratio in reaction buffer containing 5 mM Mn^{2+} ions for 1 h at 37 °C. All cleavage experiments were carried out in triplicates.

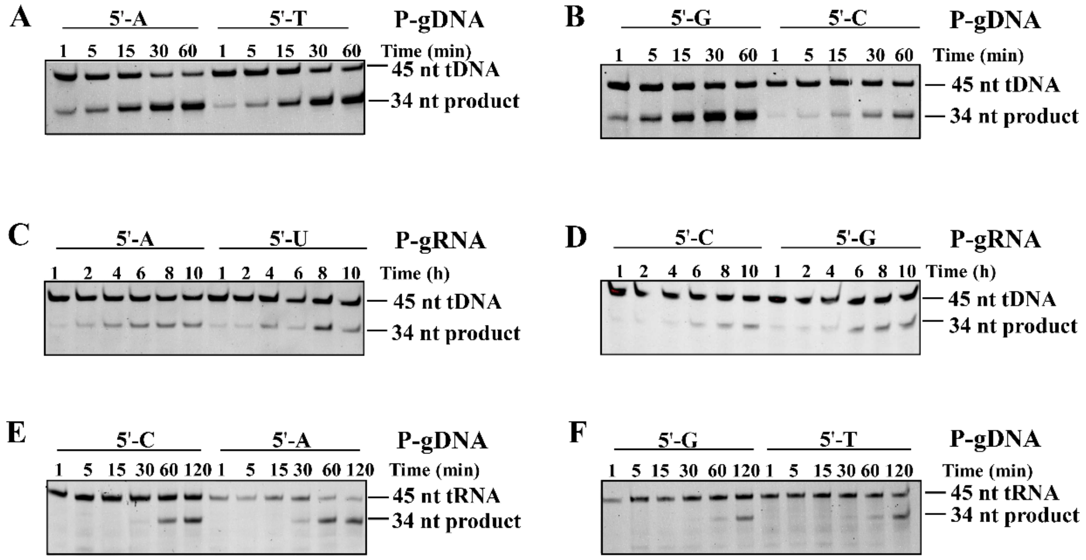

Figure S6. Effect of 5'-end nucleotide on DNA target cleavage activity. (**A**) Effects of the 5'-A and 5'-T terminal nucleotides of P-gDNA on DNA target cleavage activity. (**B**) Effects of the 5'-G and 5'-C terminal nucleotides of P-gDNA on DNA target cleavage activity. (**C**) Effects of the 5'-A and 5'-U terminal nucleotides of P-gRNA on DNA target cleavage activity. (**D**) Effects of the 5'-C and 5'-G terminal nucleotides of P-gRNA on DNA target cleavage activity. (**E**) Effects of the 5'-C and 5'-A terminal nucleotides of P-gDNA on RNA target cleavage activity. (**F**) Effects of the 5'-G and 5'-T terminal nucleotides of P-gDNA on RNA target cleavage activity. All experiments were performed at the 4: 2: 1 RsuAgo: guide: target molar ratio in reaction buffer containing 5 mM Mn^{2+} ions at 37 °C. All cleavage experiments were carried out in triplicates.

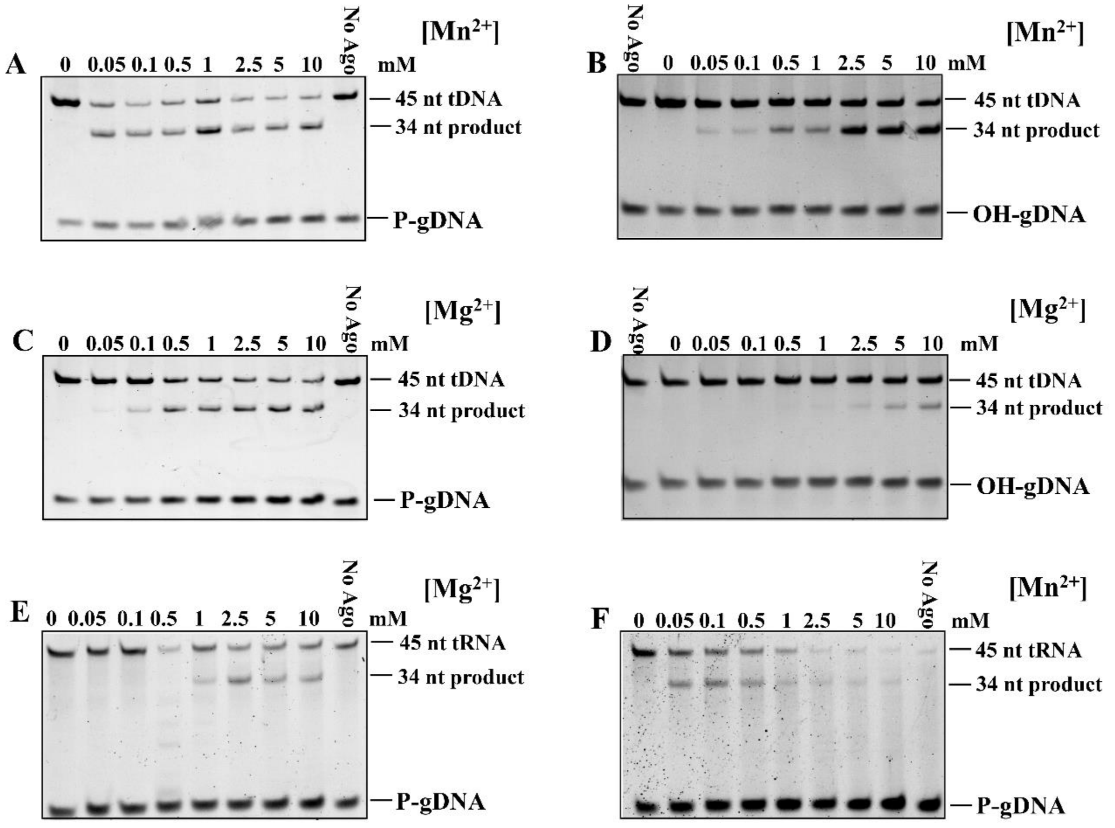

Figure S7. Effects of Mn^{2+} and Mg^{2+} concentration on DNA and RNA target cleavage activity. (**A**) Effects of Mn^{2+} concentration on DNA target cleavage guided by P-gDNA. (**B**) Effects of Mn^{2+} concentration on DNA target cleavage guided by OH-gDNA. (**C**) Effects of Mg^{2+} concentration on DNA target cleavage guided by P-gDNA. (**D**) Effects of Mg^{2+} concentration on DNA target cleavage guided by OH-gDNA. (**E**) Effects of Mg^{2+} concentration on RNA target cleavage guided by P-gDNA. (**F**) Effects of Mn^{2+} concentration on RNA target cleavage guided by P-gDNA. All experiments were performed at the 4: 2: 1 RsuAgo: guide: target molar ratio in reaction buffer containing different divalent metal ions for 1 h at 37 °C. All cleavage experiments were carried out in triplicates.

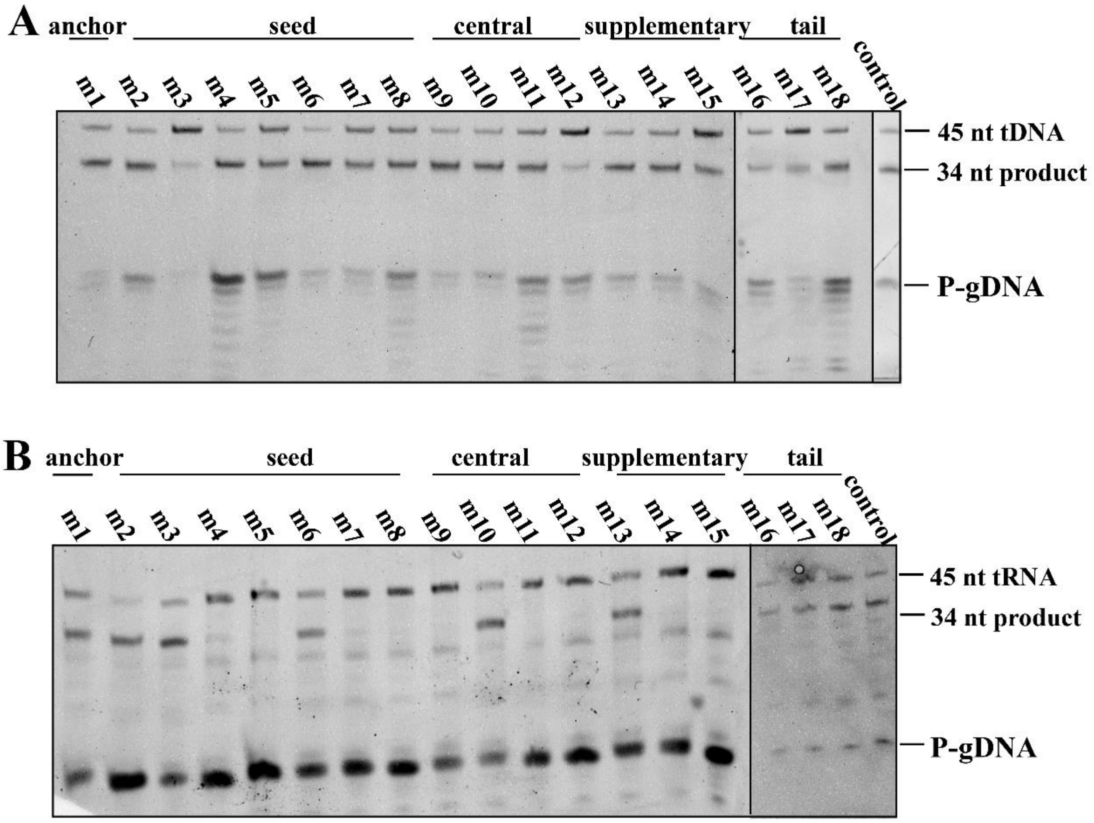

Figure S8. Effects of mismatches on the cleavage activity of RsuAgo. (**A**) Effects of single nucleotide mismatches in the P-gDNA: tDNA duplex on the slicing activity of RsuAgo. (**B**) Effects of single nucleotide mismatches in the P-gDNA: tRNA duplex on the slicing activity of RsuAgo. Control, gDNA with full complementarity to the target. All experiments were performed at the 4: 2: 1 RsuAgo: guide: target molar ratio in reaction buffer containing 5 mM Mn^{2+} ions for 1 h at 37 °C. All cleavage experiments were carried out in triplicates.

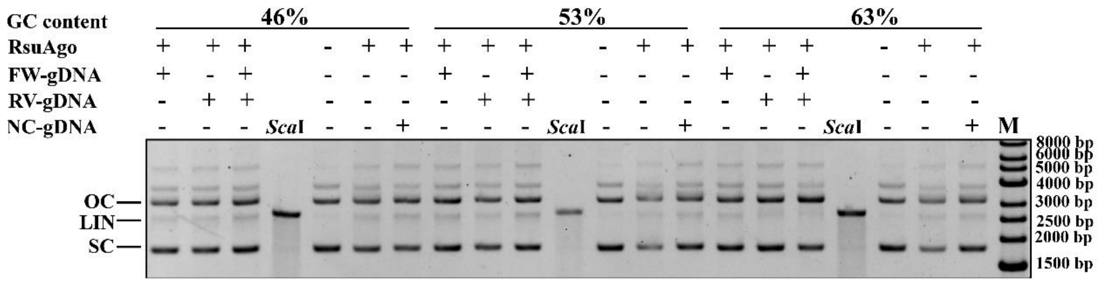

Figure S9. Double-stranded plasmid DNA cleavage by RsuAgo. **A** RsuAgo-mediated plasmid pUC19 cleavage with gDNAs differing in their GC-content in reaction buffer containing 0.5 mM

Mn^{2+} and 55 mM NaCl for 2 h at 37 °C. *Sca*I lane: the isolated plasmid was digested with *Sca*I for 2 h. FW/RV-gDNA, forward and reverse gDNA corresponding to a specific target site in plasmid pUC19; NC-gDNA, a non-complementary gDNA control; M, molecular weight marker; LIN, linearized plasmid; SC, supercoiled plasmid; OC, open circular plasmid. All cleavage experiments were carried out in triplicates.

Table S1. List of let-7-derived sequences used in this study.

Oligonucleotide name	Sequence (5'-3')	Description
FAM-tDNA	FAM-AAACGACGGCCAGTGCCAAGCTTACTATACAACCTACTACCTCAT	5' FAM labeled T-tDNA
M1	AAACGACGGCCAGTGCCAAGCTTACTATACAACC	5' no FAM labeled 34-nt DNA
FAM-M1	FAM-AAACGACGGCCAGTGCCAAGCTTACTATACAACC	5' FAM labeled 34-nt DNA
FAM-tRNA	FAM-AAACGACGGCCAGUGCCAAGCUUACUAUACAACCUACUACCUCAU	5' FAM labeled U-tRNA
M2	AAACGACGGCCAGUGCCAAGCUUACUAUACAACC	5' no FAM labeled 34-nt RNA
FAM-M2	FAM-AAACGACGGCCAGUGCCAAGCUUACUAUACAACC	5' FAM labeled 34-nt RNA
C-gDNA	CGAGGTAGTAGGTTGTAT	guide forms 5'-C pair with C-tDNA/C-tRNA
T-gDNA	TGAGGTAGTAGGTTGTAT	guide forms 5'-T pair with T-tDNA/T-tRNA
A-gDNA	AGAGGTAGTAGGTTGTAT	guide forms 5'-A pair with A-tDNA/A-tRNA
G-gDNA	GGAGGTAGTAGGTTGTAT	guide forms 5'-G pair with G-tDNA/G-tRNA
A-tDNA	AAACGACGGCCAGTGCCAAGCTTACTATACAACCTACTACCTCTT	let-7 based 45-nt DNA target for A-gDNA/A-gRNA
G-tDNA	AAACGACGGCCAGTGCCAAGCTTACTATACAACCTACTACCTCCT	let-7 based 45-nt DNA target for G-gDNA/G-gRNA
C-tDNA	AAACGACGGCCAGTGCCAAGCTTACTATACAACCTACTACCTCGT	let-7 based 45-nt DNA target for C-gDNA/C-gRNA

T-tDNA	AAACGACGGCCAGTGCCAAGCTT ACTATACAACCTACTACCTCAT	let-7 based 45-nt DNA target for T-gDNA/U-gRNA
34-nt DNA product	AAACGACGGCCAGTGCCAAGCTT ACTATACAACC	5' DNA product for 45-nt DNA target
C-gRNA	CGAGGUAGUAGGUUGUAU	guide forms 5'-C pair with C-tDNA/C-tRNA
U-gRNA	UGAGGUAGUAGGUUGUAU	guide forms 5'-U pair with T-tDNA/T-tRNA
A-gRNA	AGAGGUAGUAGGUUGUAU	guide forms 5'-A pair with A-tDNA/A-tRNA
G-gRNA	GGAGGUAGUAGGUUGUAU	guide forms 5'-G pair with G-tDNA/G-tRNA
C-tRNA	AAACGACGGCCAGUGCCAAGCUU ACUAUACAACCUACUACCUCGU	let-7 based 45-nt RNA target for C-gRNA/C-gDNA
U-tRNA	AAACGACGGCCAGUGCCAAGCUU ACUAUACAACCUACUACCUCAU	let-7 based 45-nt RNA target for U-gDNA/U-gRNA
A-tRNA	AAACGACGGCCAGUGCCAAGCUU ACUAUACAACCUACUACCUCUU	let-7 based 45-nt RNA target for A-gDNA/A-gRNA
G-tRNA	AAACGACGGCCAGUGCCAAGCUU ACUAUACAACCUACUACCUCCU	let-7 based 45-nt RNA target for G-gDNA/G-gRNA
34-nt RNA product	AAACGACGGCCAGUGCCAAGCUU ACUAUACAACC	5' RNA product for 45-nt RNA target
gDNA_m1	TGAGGTAGTAGGTTGTAT	guide forms mismatched pair in position 1 with A-tDNA/A-tRNA
gDNA_m2	ACAGGTAGTAGGTTGTAT	guide forms mismatched pair in position 2 with A-tDNA/A-tRNA
gDNA_m3	AGTGGTAGTAGGTTGTAT	guide forms mismatched pair in position 3 with A-tDNA/A-tRNA
gDNA_m4	AGACGTAGTAGGTTGTAT	guide forms mismatched pair in position 4 with A-tDNA/A-tRNA
gDNA_m5	AGAGCTAGTAGGTTGTAT	guide forms mismatched pair in position 5 with A-tDNA/A-tRNA
gDNA_m6	AGAGGAAGTAGGTTGTAT	guide forms mismatched pair in position 6 with A-

		tDNA/A-tRNA
gDNA_m7	AGAGGTTGTAGGTTGTAT	guide forms mismatched pair in position 7 with A-tDNA/A-tRNA
gDNA_m8	AGAGGTACTAGGTTGTAT	guide forms mismatched pair in position 8 with A-tDNA/A-tRNA
gDNA_m9	AGAGGTAGAAGGTTGTAT	guide forms mismatched pair in position 9 with A-tDNA/A-tRNA
gDNA_m10	AGAGGTAGTTGGTTGTAT	guide forms mismatched pair in position 10 with A-tDNA/A-tRNA
gDNA_m11	AGAGGTAGTACGTTGTAT	guide forms mismatched pair in position 11 with A-tDNA/A-tRNA
gDNA_m12	AGAGGTAGTAGCTTGTAT	guide forms mismatched pair in position 12 with A-tDNA/A- tRNA
gDNA_m13	AGAGGTAGTAGGATGTAT	guide forms mismatched pair in position 13 with A-tDNA/A-tRNA
gDNA_m14	AGAGGTAGTAGGTAGTAT	guide forms mismatched pair in position 14 with A-tDNA/A-tRNA
gDNA_m15	AGAGGTAGTAGGTTCTAT	guide forms mismatched pair in position 15 with A-tDNA/A-tRNA
gDNA_m16	AGAGGTAGTAGGTTGAAT	guide forms mismatched pair in position 16 with A-tDNA/A-tRNA
gDNA_m17	AGAGGTAGTAGGTTGTTT	guide forms mismatched pair in position 17 with A-tDNA/A-tRNA
gDNA_m18	AGAGGTAGTAGGTTGTAA	guide forms mismatched pair in position 18 with A-tDNA/A-tRNA
8-nt A-gDNA	AGAGGTAG	8-nt guide pair with A-tDNA/A-tRNA
9-nt A-gDNA	AGAGGTAGT	9-nt guide pair with A-tDNA/A- tRNA
10-nt A-gDNA	AGAGGTAGTA	10-nt guide pair with A-tDNA/A-tRNA
11-nt A-gDNA	AGAGGTAGTAG	11-nt guide pair with A-

		tDNA/A-tRNA
12-nt A-gDNA	AGAGGTAGTAGG	12-nt guide pair with A-tDNA/A-tRNA
13-nt A-gDNA	AGAGGTAGTAGGT	13-nt guide pair with A-tDNA/A-tRNA
14-nt A-gDNA	AGAGGTAGTAGGTT	14-nt guide pair with A-tDNA/A-tRNA
15-nt A-gDNA	AGAGGTAGTAGGTTG	15-nt guide pair with A-tDNA/A-tRNA
16-nt A-gDNA	AGAGGTAGTAGGTTGT	16-nt guide pair with A-tDNA/A-tRNA
17-nt A-gDNA	AGAGGTAGTAGGTTGTA	17-nt guide pair with A-tDNA/A-tRNA
18-nt A-gDNA	AGAGGTAGTAGGTTGTAT	18-nt guide pair with A-tDNA/A-tRNA
19-nt A-gDNA	AGAGGTAGTAGGTTGTATA	19-nt guide pair with A-tDNA/A-tRNA
20-nt A-gDNA	AGAGGTAGTAGGTTGTATAG	20-nt guide pair with A-tDNA/A-tRNA
21-nt A-gDNA	AGAGGTAGTAGGTTGTATAGT	21-nt guide pair with A-tDNA/A-tRNA
25-nt A-gDNA	AGAGGTAGTAGGTTGTATAGTAAGC	25-nt guide pair with A-tDNA/A-tRNA
30-nt A-gDNA	AGAGGTAGTAGGTTGTATAGTAAGCTTGGC	30-nt guide pair with A-tDNA/A-tRNA
40-nt A-gDNA	AGAGGTAGTAGGTTGTATAGTAAGCTTGGCACTGGCCGTC	40-nt guide pair with A-tDNA/A-tRNA

Table S2. List of gDNAs targeting plasmid pUC19.

gDNA	Sequence (5′-3′)	GC-content of the target region (%)
29GC-F	TCAAAAAGGATCTTCACC	29
29GC-R	TAGGTGAAGATCCTTTTT	29
39GC-F	AAGAAACCATTATTATCA	39
39GC-R	CATGATAATAATGGTTTC	39
45GC-F	AAAAGTGCTCATCATTGG	45
45GC-R	TTCCAATGATGAGCACTT	45
46GC-F	CAACATACGAGCCGGAAG	46
46GC-R	TGCTTCCGGCTCGTATGT	46
53GC-F	CAGGGTTTTCCCAGTCAC	53
53GC-R	TCGTGACTGGGAAAACCC	53
63GC-F	CGCTCGGTCGTTCGGCTG	63
63GC-R	CGCAGCCGAACGACCGAG	63
